# Childhood Maltreatment and Immune Cell Gene Regulation during Adolescence: Transcriptomics Highlight Non-Classical Monocytes

**DOI:** 10.3390/biom14020220

**Published:** 2024-02-13

**Authors:** Kate R. Kuhlman, Steve W. Cole, Ece N. Tan, James A. Swanson, Uma Rao

**Affiliations:** 1 Department of Psychological Science, School of Social Ecology, University of California Irvine, 4546 Social & Behavioral Sciences Gateway, Irvine, CA 92697, USA; 2 Cousins Center for Psychoneuroimmunology, Semel Institute for Neuroscience and Human Behavior, University of California Los Angeles, Los Angeles, CA 90095, USA; 3 Department of Psychiatry & Human Behavior, School of Medicine, University of California Irvine, Irvine, CA 92697, USA; 4 Stritch School of Medicine, Loyola University Chicago, Maywood, IL 60153, USA; 5 Center for the Neurobiology of Learning and Memory, University of California Irvine, Irvine, CA 92697, USA; 6 Children’s Hospital of Orange Country (CHOC), Orange, CA 92868, USA

**Keywords:** maltreatment, adolescent, psychoneuroimmunology, non-classical CD16+ monocytes, IRF, NRF1, MAF

## Abstract

Childhood maltreatment has been repeatedly linked to a higher incidence of health conditions with an underlying proinflammatory component, such as asthma, chronic obstructive pulmonary disease, stroke, and cardiovascular disease. Childhood maltreatment has also been linked to elevated systemic inflammation prior to the onset of disease. However, childhood maltreatment is highly comorbid with other risk factors which have also been linked to inflammation, namely major depression. The present analysis addresses this issue by assessing the association of maltreatment with genome-wide transcriptional profiling of immune cells collected from four orthogonal groups of adolescents (aged 13–17): maltreated and not maltreated in childhood, with and without major depressive disorder. Maltreatment and psychiatric history were determined using semi-structured clinical interviews and cross-validated using self-report questionnaires. Dried whole blood spots were collected from each participant (*n* = 133) and assayed to determine the extent to which maltreatment in childhood was associated with a higher prevalence of transcriptional activity among differentially expressed genes, specific immune cell subtypes, and up- or down-regulation of genes involved in immune function after accounting for current major depression. Maltreatment was associated with increased interferon regulatory factor (IRF) transcriptional activity (*p* = 0.03), as well as nuclear factor erythroid-2 related factor 1 (NRF1; *p* = 0.002) and MAF (*p* = 0.01) among up-regulated genes, and increased activity of nuclear factor kappa beta (NF-κB) among down-regulated genes (*p* = 0.01). Non-classical CD16+ monocytes were implicated in both the up- and down-regulated genes among maltreated adolescents. These data provide convergent evidence supporting the role of maltreatment in altering intracellular and molecular markers of immune function, as well as implicate monocyte/macrophage functions as mechanisms through which childhood maltreatment may shape lifelong immune development and function.

## 1. Introduction

One in seven children in the U.S. are maltreated each year through exposure to physical or emotional neglect and physical, emotional, or sexual abuse [1]. The economic burden of childhood maltreatment is estimated to be an additional $210,012 per individual across their lifespan and a total of at least $428 billion annually [2]. Adults, particularly women, who were maltreated as children are at greater risk for a wide range of diseases including neurovascular disease (e.g., stroke), cardiovascular disease, asthma, and chronic obstructive pulmonary disease [3,4,5], as well as earlier all-cause mortality [6]. The potentially modifiable mechanisms linking childhood maltreatment to these lifelong health risks remains unknown, and must be elucidated if these trajectories are to be mitigated. 

One putative pathway linking maltreatment in childhood with lifelong susceptibility to illness is through biological embedding of experience during sensitive phases of development that lead to persistent changes to physiological systems involved in adapting to the environment, including the immune system [7,8,9,10]. Specifically, the innate immune system is responsible for the fast and non-specific reaction to potential pathogens that is largely organized through the mobilization of different types of immune cells via secretion of proteins (i.e., cytokines and chemokines). Studies have linked early life adversity (including, but not limited to, maltreatment) to elevated concentrations of these proteins in circulating blood among adolescents [11] and adults [12,13], and this pattern tends to increase across the lifespan [14]. Inflammation is mediated by innate immune cells and the molecules they release (e.g., cytokines, chemokines, and their physiological byproducts in circulation). Inflammation contributes directly to the pathogenesis of disease (c.f. cancer: [15], vascular disease: [16]). For this reason, a mechanistic understanding of the ways in which maltreatment and other common forms of adversity exposure during childhood influence the immune system is needed.

Studies have shown that immune cells from individuals exposed to chronic stress during childhood may produce more cytokines and chemokines in response to in vitro stimulation with a pathogen [14] and activate more genes involved in pro-inflammatory signaling in response to acute psychosocial stress [17,18]. This suggests that adversity during childhood, such as maltreatment, may alter the sensitivity of immune cells to the environment and their propensity to activate or initiate communication with the rest of the immune system. Some transcription factors, which drive immune cell signaling, have been implicated in the differential immune activity among maltreatment-exposed individuals. For example, among adult women with breast cancer, childhood maltreatment has been associated with greater transcriptional activity of the nuclear factor kappa-light-chain-enhancer of activated B cells [NF-κB] [18,19,20] and the interferon regulatory factors (IRF) [19], suggesting a pattern of exaggerated immune activation and propensity for proinflammatory signaling. A similar pattern [using a composite of interleukin (IL)-1B, IL-8, and prostaglandin-endoperoxide synthase (PTGS2) gene activity] has also been observed among older adults exposed to childhood maltreatment relative to those who were not [21].

Exaggerated immune activation has been observed among adversity-exposed children and adolescents as well. For example, among school-aged children, each additional adversity exposure was associated with greater expression of both proinflammatory and type 1 interferon (IFN; anti-viral) genes [22]. When stimulated in vitro, lymphocytes from maltreatment-exposed adolescents produced more cytokines [IL-2, IL-4, interferon-γ, and IL-17] and evidenced more transcriptional activity associated with cytokine production including NF-κB [23]. Similarly, more exposure to different types of social adversity during childhood was associated with greater production of cytokines as well as JAK-STAT and NF-κB activity following in vitro stimulation [24]. Following standardized psychosocial stress in a laboratory, adolescents exposed to more adversity showed greater activation of both proinflammatory and anti-viral genes, as well as an elevated cAMP response element-binding protein (CREB) transcriptional activity, which plays a key role in adrenergic signaling pathways that can subsequently stimulate inflammatory activity [25]. Taken together, early life adversity has been associated with immune cells that have a propensity to produce more cytokines as a by-product of greater IRF, NF-κB, and stress-induced CREB transcriptional activity.

Defining the specific type of leukocyte involved may help clarify the biological mechanisms of increased health risk in children subjected to early life adversity. Monocytes are a critical cell of the innate immune system and show a great deal of functional plasticity depending on their immediate microenvironment [26,27]. In the absence of a pathogen or injury, monocytes are relatively quiescent and contribute to homeostatic tissue maintenance; indeed, their quiescent role in the body has been described as largely “janitorial”, clearing away old red blood cells and cells that have undergone apoptosis, as well as repairing damaged tissue throughout the body [27]. There are several external factors that can lead to a functional shift in monocytes/macrophages for the purpose of host defense, wound healing, or immune regulation. These three purposes are achieved through three functional phenotypes of activated monocytes/macrophages, which are induced by the presence of cytokines and transcriptional activity. For example, NF-κB and IRF have both been associated with macrophage activation in favor of host defense against pathogens, which involves killing and ingesting foreign cells [27,28]. Notably, there is converging evidence in non-human primates [8], children [22], adolescents [25], and adults [18,19] that the proinflammatory phenotype associated with early life adversity is associated with non-classical CD16+ monocytes. Non-classical CD16+ monocytes are a minority subpopulation of monocytes that are more differentiated and mature than their classical counterparts, are more likely to have migrated into tissue and lymphatic vessels, and often proliferate into dendritic cells [29,30]. The mechanisms through which differential gene expression observed among maltreatment-exposed individuals is associated with the non-classical CD16+ monocyte phenotype are largely unknown. Resolving this gap in our understanding may provide a more specific cellular basis for understanding how childhood maltreatment portends increasing inflammation across the lifespan.

One of the most prominent limitations to understanding the links between maltreatment and inflammatory biology in humans is that maltreatment is confounded by major depressive disorder (MDD) in community samples. For example, some of our best epidemiological data suggest that 59% of children with a mood disorder have a history of maltreatment or chronic adversity [31]. Across the lifespan, individuals exposed to maltreatment are at 4-fold greater risk for MDD [31,32,33,34]. This is particularly true when looking at immune mechanisms because adversity is associated with increasing peripheral markers of inflammation in adolescence [11,14], and individuals with MDD often evidence elevated markers of inflammation both centrally [35] and peripherally [36,37,38,39]. Notably, this coupling of inflammation and depression is already apparent during adolescence [40]. Taken together, making inferences that can be drawn about the specific role of adversity that is independent of the experience of having MDD can seem impossible. As a result, whether depression and its symptoms are the mechanism through which childhood maltreatment and broader forms of early life adversity lead to increased inflammation, morbidity, and earlier mortality or whether maltreatment independently advances these outcomes has remained unclear.

This study sought to determine the association between childhood maltreatment and inflammatory biology using genome-wide transcriptional profiling of immune cells in a sample recruited with the explicit goal of disentangling maltreatment from MDD. To do so, four orthogonal groups of adolescents were recruited: those with moderate-to-severe childhood maltreatment and current MDD (MALTX + MDD), those with only current MDD (MDD only) or severe childhood maltreatment (MALTX only), or those with neither MDD nor childhood maltreatment (CTL). We hypothesized that, independent of MDD, childhood maltreatment would be associated with differential gene expression that disproportionately originated within non-classical CD16+ monocytes relative to other immune cell subtypes, differential NF-κB, IRF, and CREB transcriptional activity, and enhanced activation of genes with known involvement in proinflammatory immunity.

## 2. Materials and Methods

### 2.1. Participants

This analysis represents data from 133 adolescents (*M*_age_ = 15.42 ± 1.47; 75.9% female; *M* ± *SD* Tanner Stage = 4.26 ± 0.73); 47.4% of this sample identified as white, 25.6% as multiracial, 12.8% as black or African American, 9% as Asian, 4.5% as American Indian or Alaska Native, and 0.8% as Native Hawaiian or other Pacific Islander. Approximately half (50.4%) identified their ethnicity as Hispanic or Latino.

These 133 adolescents represented 86.4% of the adolescents who were enrolled to date in a larger ongoing study [41] and were asked to provide a dried blood spot (DBS) as part of a supplemental study. The supplemental DBS collection was added to the study’s protocol after enrollment of the first fourteen participants, and subsequent participants were free to consent or decline participation in the DBS collection.

Inclusion criteria for the larger study were male and female youth aged 13–17 years at the time of enrollment, and Tanner Stage ≥ 2. Exclusion criteria included a clinically significant neurological history, an IQ below 80, diagnosis of autism spectrum disorder, psychotic disorders or a prior history of mania or hypomania in the adolescent or first-degree relatives, MRI contraindications (e.g., nonremovable metal inside or outside of the body or claustrophobia), pregnancy, recent use of certain psychotropic medications including antidepressants, use of alcohol or drugs in the week leading up to enrollment in the study, and a history of multiple unrelated forms of adversity (e.g., prolonged separation/loss of primary caretaker, house fire, or war). Exclusion criteria were evaluated via open-ended questions about the prospective participant’s medical history such as “Have you ever received a diagnosis for your child?”, “Is your child on any medication?”, “Has your child ever had any major medical problems?”, “Has your child ever lost consciousness?” Responses were reviewed on a case-by-case basis by the principal investigator who is a psychiatrist. For example, individuals were excluded from participation for neurological reasons if their condition was likely to interfere with their ability to participate in the study or if they were currently being treated pharmacologically for the condition. Specific reasons that led to exclusion from this study included neurofibromatosis type 1, pseudotumor condition, Arnold Chiari Malformation Grade I, frontal lobe cysts, and epilepsy.

### 2.2. Procedures

All study procedures were approved by the Institutional Review Board at the University of California, Irvine (Protocol #2017-3440). Adolescents were recruited through organizations across Southern California, including religious and cultural organizations and those serving minority, underserved, and vulnerable youth, dissemination of flyers at fairs, social media advertisements, referrals from a local children’s hospital, other studies within the lab, and those conducted by collaborators and word of mouth.

Interested parents and their adolescents underwent phone screening to collect demographic information as well as information pertaining to the teen’s physical and mental health, his/her biological parents’ mental health history, possible MRI contraindications, and possible adverse experiences within the home environment (i.e., whether the teen ever lived with a caregiver that frequently swore at, insulted, or humiliated him/her, whether the teen ever witnessed domestic violence, and whether there was ever contact with Child Protective Services). Those deemed eligible based on this preliminary screen were then invited to enroll in the study.

During the first of three study visits, parents and adolescents provided their written informed consent and assent. They then completed a series of questionnaires and interviews, including the Kiddie Schedule for Affective Disorders and Schizophrenia K-SADS-COMP; [42] and Childhood Adversity Interview CAI; [43]. Information gathered through the administration of the KSADS and CAI allowed for the categorization of adolescents into one of the following four orthogonal groups. History of childhood maltreatment and current MDD (MALTX + MDD): This group included adolescents who met DSM-5 criteria for current MDD based on the KSADS and experienced maltreatment before the age of 10 based on a rating of 3 or higher on the physical, emotional, or sexual abuse/assault categories of the CAI. Current MDD only (MDD only): This group included adolescents who met criteria for current MDD based on the KSADS and received a rating of less than 2 on each maltreatment item on the CAI, including emotional and physical neglect and domestic violence, indicating no history of maltreatment. History of childhood maltreatment only (MALTX only): This group included adolescents who experienced maltreatment according to the criteria above, without a history of any psychiatric disorder, with the exception of phobias. Neither MDD nor a history of childhood maltreatment (CTL): This group included adolescents who had no personal history of, or a first-degree relative with, a psychiatric disorder, except phobias, based on the KSADS and Family History-Research Diagnostic Criteria [44], respectively, as well as no history of maltreatment. Adolescents who did not meet criteria for one of these four groups were excluded from participation.

The vast majority of adolescents were also asked to provide a DBS sample during the initial study visit. For a small number of participants, DBS collection occurred during a subsequent visit (within 15 days) due to time constraints. Prior to collection, adolescents were provided water to drink and instructed to remain seated for at least 30 min. The participant’s non-dominant hand was positioned at or below the elbow, a warm gel pack was placed on the hand for five minutes, and then the hand was massaged to promote blood flow. The participant’s middle finger was sterilized with an alcohol swab, and the tip of the finger was punctured with a lancet. The first drop of blood that was collected at the puncture site was wiped away with an alcohol swab; subsequent drops of blood were allowed to fall naturally onto a Whatman #10 DBS collection card until the five circles on the card were filled completely or the puncture site clotted. If an insufficient amount of blood was collected, the participant was asked if they consent to a second puncture on their ring finger. The collection card was kept open in a light-protected drying container overnight, at room temperature. After the spots were completely dry and within one week of collection, the card was closed, placed in a storage bag with a desiccant pack, and stored in a container under the same conditions until transfer to a −80 °C freezer. Participants were compensated an additional $15 for attempting DBS collection.

### 2.3. Measures

Maltreatment. Maltreatment exposure was determined using the CAI [43]. Both parents and their teens separately completed the semi-structured interview with a trained staff member, who was under the supervision of a licensed, doctoral-level clinician. A comprehensive history of any: 1. separation with/loss of a caretaker; 2. life-threatening injury or illness in self, parent/caretaker, or sibling or loss of a sibling, other relative, or close friend; 3. physical neglect; 4. emotional abuse/assault; 5. physical abuse/assault, 6. witnessing domestic violence and; 7. sexual abuse/assault occurring prior to and after the adolescent participant’s tenth birthday was obtained. Information regarding the timing, severity, duration, and frequency of these events was also collected. The interviewer then rated each informant’s response on a scale of 1 to 5 (with half scores possible; 1 = no adversity, 3 = moderate, and 5 = extreme) for incidents prior to age 10 as well as lifetime, and determined a final severity rating for each of the seven categories.

Depression. Presence of current depression was assessed using a web-based semi-structured interview, K-SADS-COMP [42]. This interview can be administered to children between the ages of 6 and 17 and their parents. It is used to ascertain the presence of current psychopathology, as well as lifetime, according to DSM-5 diagnostic criteria. The modules assessed in this study were mood disorders, psychosis, panic disorder, agoraphobia, social anxiety disorder, generalized anxiety disorder, obsessive compulsive disorder, eating disorders, attention deficit hyperactivity disorder, conduct disorder, autism spectrum disorder, alcohol use disorder, drug use disorders, post-traumatic stress disorder, sleep problems, suicidality, and homicidality.

Severity of depressive symptoms was assessed using the Children’s Depression Rating Scale-Revised CDRS-R; [45], which is a semi-structured interview that can be administered to youth aged 6 to 17 years. The 17 depressive symptoms assessed included impaired school work, difficulty having fun, social withdrawal, sleep disturbance, appetite disturbance, excessive fatigue, physical complaints, irritability, excessive guilt, low self-esteem, depressed feelings, morbid ideation, suicidal ideation, excessive weeping, depressed facial affect, listless speech, and hypoactivity. Sleep and appetite disturbance and listless speech were rated on a scale of 1 to 5, and all other symptoms were rated on a scale of 1 to 7 (1 = absence of symptom; 5 or 7 = presence of severe symptoms).

Depressive symptoms were also assessed via adolescent self-report using the 21-item Beck Depression Inventory (BDI) [46]. Each item consisted of four or five statements, and adolescents were instructed to pick the statement that best described how they felt in the previous week (e.g., 0 = “I do not feel like a failure,” 1 = “I feel I have failed more than the average person,” 2 = “I feel I have accomplished very little that is worthwhile or that means anything,” 3 = “As I look back on my life, all I can see is a lot of failure,” or 4 = “I feel I am a complete failure as a person (parent, husband, wife)”). Scores on each item were added to calculate a composite score; a higher score indicated a higher severity of depressive symptoms.

Gene expression. Genome-wide transcriptional profiling was completed on DBS samples from all participants who provided blood samples. Samples were shipped on dry ice overnight to the UCLA Social Genomics Core Laboratory for RNA extraction, quality assurance, and transcriptional profiling as previously described [47]. Briefly, 2–4- blood spots were cut from each participant’s 5-spot sample. Spots were then submerged in 370 μL of RLT in an RNAse-free sterile 1.5 mL microcentrifuge tube, incubating the tube for 30 min at 37 °C with agitation (1000 rpm), and transferring tube contents (including filter paper) into a QIAshredder column for 60 s of microcentrifugation at maximum speed, after which the 360 μL of remaining RLT was processed through the QIAcube nucleic acid extraction system using RNeasy Micro Kit reagents, the manufacturer’s standard operating protocol (including DNAse treatment), and a 20 μL elution volume (QIAGEN, Hilden, Germany). RNA was then subject to genome-wide transcriptional profiling using a high efficiency mRNA-sequencing assay (Lexogen QuantSeq 3′ FWD) following the manufacturer’s standard protocol. Assays were performed in a single batch and targeted 5 million sequencing reads per sample (achieved median = 4.98 million), each of which was mapped to the GRCh38 reference human transcriptome using the STAR aligner to quantify transcript abundance (achieved median mapping rate = 97.7%). Transcript abundance data were normalized counts per million mapped reads and log2 transformed for statistical analyses [8,47,48].

### 2.4. Data Analysis

Primary analyses employed linear mixed effect models to (1) identify differential transcriptional activity among up- or down-regulated genes among adolescents categorized as maltreated or not maltreated during childhood, (2) the cellular origins of this differential transcriptional activity, and (3) to determine whether childhood maltreatment was associated with increased expression of an a priori subset of proinflammatory or antiviral immune response genes. Normalized gene expression values were transformed to log_2_ for general linear model analyses quantifying the association of transcript abundance with categorical membership of the maltreated or non-maltreated group while also controlling for participant self-reported gender, race, ethnicity, body mass index (BMI), and smoking history. Analyses also controlled for the prevalence of transcripts marking T lymphocyte subsets (*CD3D*, *CD3E*, *CD4*, *CD8A*), B lymphocytes (*CD19*), natural killer cells (*CD16*/*FCGR3A*, *CD56*/*NCAM1*), and monocytes (*CD14*) to ensure that results were not confounded by individual differences in the prevalence of specific leukocyte subtypes within the cell pool [49]. In all analyses, statistical testing of the average log-ratio’s difference from the null hypothesis value of 0 was based on standard errors derived from bootstrap resampling of linear model residual vectors, which provides a non-parametric assessment of statistical significance while appropriately controlling for correlation among genes.

Initial “low-level” genome-wide analyses identified all transcripts showing a point estimate of ≥1.5-fold differential expression across the range −2 SD to +2 SD among maltreated relative to non-maltreated youth. Those putatively associated genes were subject to transcription element listening system (TELiS) promoter-based bioinformatic transcriptional activity analyses [50] to quantify the activity of NF-κB, IRF, CREB, and macrophage activation factor (MAF) family transcription factors which have been previously linked to inflammatory biology in individuals exposed to early social adversity. Results of transcriptional activity were averaged over nine parametric variations of MatInspector scan stringency and promoter length. Transcription factor binding motif (TFBM) prevalence was quantified by the average (log-) ratio of TFBM in up- vs. down-regulated promoters across nine combinations of three core promoter sequence lengths (−300, −600, and −1000 to +200 nucleotides relative to the RefSeq transcription start site) and three TFBM detection stringencies (TRANSFAC mat_sim values of 0.80, 0.90, and 0.95). Transcript origin analysis (TOA) was applied to the same low-level association data to identify the specific cell subtypes mediating the observed differences in gene expression, as previously described [51].

The a priori-defined proinflammatory genes examined included: *IL1A*, *IL1B*, *IL6*, *IL8*, *TNF*, *PTGS1*, *PTGS2*, *FOS*, *FOSB*, *FOSL1*, *FOSL2*, *JUN*, *JUNB*, *JUND*, *NFKB1*, *NFKB2*, *REL*, *RELA*, and *RELB*. The a priori “antiviral” genes involved in type I IFN responses and antibody synthesis included: *GBP1*, *IFI16*, *IFI27*, *IFI27L1-2*, *IFI30*, *IFI35*, *IFI44*, *IFI44L*, *IFI6*, *IFIH1*, *IFIT1-3*, *IFIT5*, *IFIT1L*, *IFITM1-3*, *IFITM4P*, *IFITM5*, *IFNB1*, *IRF2*, *IRF7-8*, *MX1-2*, *OAS1-3*, *OASL, IGJ*, *IGLL1*, and *IGLL3* [48,52].

## 3. Results

Participant characteristics are summarized in Table 1. Participants were predominantly female, racially and ethnically diverse. With respect to childhood maltreatment, 34.6% of participants were classified as maltreatment-exposed. These participants were less likely to be white, *d* = 0.06 (*SE* = 0.07), *p* = 0.39, *X*^2^ =11.95, *p* = 0.04, and more likely to meet criteria for depression, *d* = 0.22 (*SE* = 0.09), *p* = 0.01, *X*^2^ = 6.12, *p* = 0.01, but not more likely to be male or female, *d* = 0.04 (*SE* = 0.10), *p* = 0.01, *X*^2^ = 0.16, *p* = 0.69, Hispanic/Latino, *d* = −0.09 (*SE* = 0.08), *p* = 0.30, *X*^2^ = 1.06, *p* = 0.30, and evidenced no differences in age [*F*(1,131) = 0.33, *p* = 0.57] or Tanner stage [*F*(1,131) = 0.79, *p* = 0.38]. Importantly, depressive symptom severity did not differ between the depressed participants in the sample who did and did not have a history of maltreatment [CDRS: *F*(1,74) = 2.71, *p* = 0.10; BDI: *F*(1,74) = 0.26, *p* = 0.61]. However, among the maltreated participants, maltreatment severity as measured by self-report was greater among the depressed, relative to the non-depressed participants [CTQ: *F*(1,44) = 5.78, *p* = 0.02].

### 3.1. Maltreatment and PBMC Gene Regulation

After adjusting for gender, race, ethnicity, BMI, and smoking history, as well as the leukocyte subsets associated with transcription factor abundance, there were 451 identified gene transcripts that differed in average expression level by >50% as a function of maltreatment exposure (109 up-regulated and 342 down-regulated). 

Initial analyses of cellular origins implicated multiple myeloid lineage cell types in the transcriptional up-regulation associated with maltreatment history, including both classical monocytes (mean TOA log diagnosticity score = 0.32 ± 0.09, *p* = 0.0002) and non-classical monocytes (mean TOA log diagnosticity score = 0.31 ± 0.06, *p* < 0.0001), as well as neutrophils (mean TOA log diagnosticity score = 0.48 ± 0.26, *p* = 0.035). Genes down-regulated in maltreated adolescents derived preferentially from type 1 dendritic cells (mean TOA log diagnosticity score = 0.10 ± 0.04, *p* = 0.003), classical monocytes (mean TOA log diagnosticity score = 0.26 ± 0.06, *p* < 0.0001), and non-classical monocytes (mean TOA log diagnosticity score = 0.26 ± 0.05, *p* < 0.0001). Given these indications of differential cellular contribution, we controlled for cell subset abundance in all analyses of transcription factor activity (to prevent misinterpretation of cell population differences as differences in per-cell signaling activity).

When accounting for covariates as well as transcript abundance among leukocyte subsets, there was a reduced prevalence of both NF-κB binding motifs within the promoters of genes up-regulated in association with maltreatment [*V$NFKAPPAB_01*: mean 0.54-fold asymmetry (*SE* = 1.19), *p* = 0.0008. By contrast, there was a greater prevalence of CREB binding motifs within the promoters of up-regulated genes associated with maltreatment [*V$CREB_Q2*: mean 1.61-fold asymmetry (*SE* = 1.20), *p* = 0.009]. There was also evidence of transcriptional activity that has yet to be reported in the literature on maltreatment, including increased MAF activity [*V$MAF_Q6_01*: mean 1.88-fold asymmetry (*SE* = 1.22), *p* = 0.002].

Consistent with the reduced activity of NF-κB and IRF signaling pathways, maltreatment was also associated with a non-significant trend toward reduced expression of 34 a priori-selected antiviral and antibody genes (*b* = −0.22, *SE* = 0.13, *p* = 0.10), and reduced expression of 19 a priori-selected proinflammatory genes that was statistically non-significant (*b* = −0.12, *SE* = 0.15, *p* = 0.42).

### 3.2. Maltreatment and PBMC Gene Expression Independent of Major Depression

We then conducted analyses to determine whether the effects of childhood maltreatment on PBMC gene regulation reported above were consistent when additionally accounting for MDD. This analysis identified 605 gene transcripts that were differentially expressed by >50% as a function of maltreatment (95 up-regulated and 510 down-regulated).

**Figure 1:**
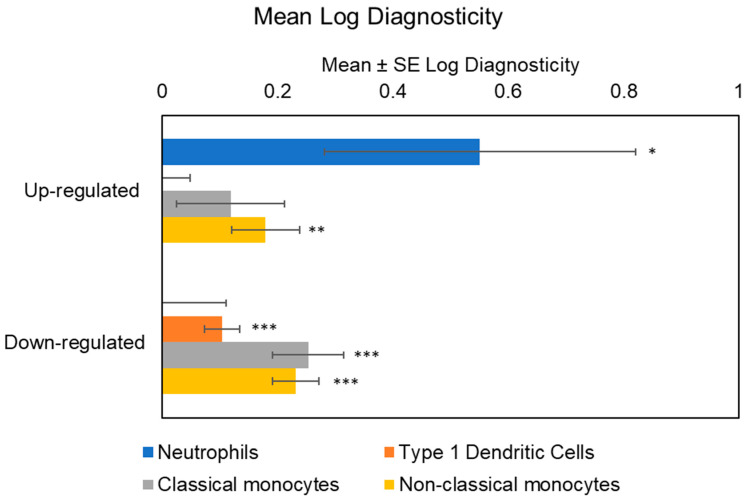
Transcript origin analyses identified genes up-regulated in maltreated adolescents’ PBMCs as originating disproportionately from neutrophils and non-classical CD16+ monocytes, while down-regulated genes originating from type 1 dendritic cells, classical CD16− monocytes, and non-classical CD16+ monocytes. Note: * *p* < 0.05, ** *p* < 0.01, *** *p* < 0.001.

Accounting for MDD abolished the bioinformatic indication of classical monocytes as contributing to transcriptional up-regulation among those with a history of maltreatment (mean TOA log diagnosticity score = 0.12 ± 0.09, *p* = 0.10), but results continued to indicate a significant contribution from non-classical monocytes (mean TOA log diagnosticity score = 0.18 ± 0.06, *p* = 0.002) and neutrophils (mean TOA log diagnosticity score = 0.55 ± 0.27, *p* = 0.02). Maltreatment-associated down-regulated genes also continued to derive from type 1 dendritic cells (mean TOA log diagnosticity score = 0.10 ± 0.03, *p* = 0.0003), classical monocytes (mean TOA log diagnosticity score = 0.25 ± 0.06, *p* < 0.0001) and non-classical monocytes (mean TOA log diagnosticity score = 0.23 ± 0.04, *p* < 0.0001). See Figure 1 for the cellular origins of differential transcriptional activity within up-and down-regulated genes among maltreated adolescents.

**Figure 2:**
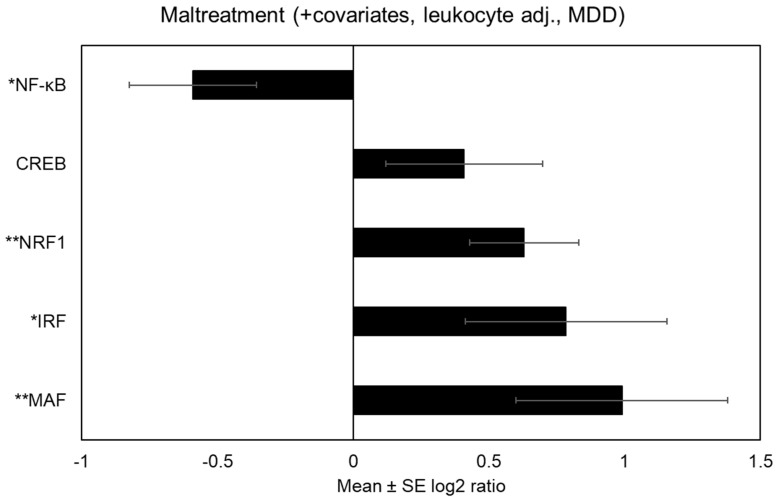
Ratio of prevalence of transcription factor binding motifs among up-regulated relative to down-regulated genes among maltreated adolescents. Figure note: * *p* < 0.05 and ** *p* < 0.01.

See Figure 2 for differentially expressed immune-related transcriptional activity among maltreated adolescents after accounting for MDD. After adjusting for MDD, adolescents exposed to maltreatment showed bioinformatic indications of increased IRF activity [*V$IRF1_01*: mean 1.72-fold asymmetry (*SE* = 1.29), *p* = 0.04] and decreased NF-κB activity [*V$NFKAPPAB_01*: mean 0.66-fold asymmetry (*SE* = 1.18), *p* = 0.01]. CREB activity no longer differed significantly, but continued to trend upward among maltreated adolescents [*V$CREB_Q2*: mean 1.33-fold asymmetry (*SE* = 1.22), *p* = 0.16]. After adjusting for MDD, there continued to be differential transcriptional activity that had not previously been reported in the literature. Specifically, maltreated adolescents evidenced bioinformatic indications of increased MAF activity [*V$MAF_Q6*: mean 1.98-fold asymmetry (*SE* = 1.31), *p* = 0.01] and increased nuclear factor erythroid-2 related factor 1 (NRF1) [*V$NRF1_Q6*: mean 1.55-fold asymmetry (*SE* = 1.15), *p* = 0.002]. Table 2 provides coefficient estimates linking maltreatment to differential immune-related transcriptional activity both unadjusted and adjusted for MDD.

There continued to be no association between maltreatment history and expression of the proinflammatory gene subset (*b* = −0.19, *SE* = 0.15, *p* = 0.22), though the non-significant trend persisted for the 34 antiviral and antibody genes (*b* = −0.25, *SE* = 0.14, *p* = 0.09), such that maltreatment history biased individuals toward down-regulation of antiviral and antibody associated genes.

## 4. Discussion

The present study sought to extend current knowledge by identifying the molecular pathways relevant to inflammatory biology that were independently associated with childhood maltreatment. Childhood maltreatment was associated with differential gene expression; both up- and down-regulated genes were disproportionately associated with non-classical monocytes, while down-regulated genes were also associated with classical monocytes and type 1 dendritic cells. Up-regulated genes were associated with IRF transcriptional activity, whereas down-regulated genes were associated with NF-κB transcriptional activity. Novel transcription factors that had not been previously linked to early life adversity were also identified, including NRF1 and MAF. Contrary to hypotheses and despite the observations linking maltreatment to differential transcriptional activity within specific immune cell subsets, maltreatment was not associated with differential expression of the *a priori* subset of inflammatory or anti-viral genes. Importantly, observations were garnered using a study design with the explicit aim of disentangling the role of childhood maltreatment from MDD. The present data converges on a link between maltreatment and the activation of the monocyte cell population away from their relatively quiescent state into a non-classically activated state.

Non-classical CD16+ monocytes were a prominent cellular origin of both up- and down-regulated genes associated with maltreatment in this sample. Non-classical CD16+ monocytes have now been implicated in differential gene expression and immune reactivity among individuals exposed to early life adversity in both non-human and human populations, in both pediatric and adult populations, and using both DBS and venipuncture at rest and in response to acute psychosocial stress [8,18,19,22,25]. In the absence of a pathogen or acute threat to the immune system, monocytes are relatively quiescent. Non-classical monocytes comprise less than 15% of the total monocyte population and are functionally distinct from the majority of monocytes in circulation; non-classical monocytes are mature monocytes that are more active and known to “patrol” the vascular system for pathogens [53]. This data may indicate that childhood maltreatment is associated with a greater propensity for monocytes to transition into and remain in this more active, non-classical state. Psychosocial stress induces an increase in monocytes among adversity-exposed adolescents [25], but not the general population [54]. Thus, individuals exposed to early life adversity may be more likely to increase their monocyte population in the context of stress, and these monocytes may be predisposed to differentiate into immune cells that behave like the non-classical CD16+ subpopulation which disproportionately secrete TNF-α [55]—the cytokine most robustly linked to early life adversity among adults [12]—become senescent [56], and contribute to disease processes such as atherosclerosis [57]. This may clarify why the association between early life adversity and markers of inflammation in circulation (e.g., acute phase proteins and cytokines) are reliable but small [11,12] and increase across the lifespan [14]. 

Previous studies had implicated increased transcriptional activity of IRF [19], NF-κB [19,20], and stress-induced CREB [25] in populations exposed to childhood maltreatment or broader forms of early life adversity. The present data provide convergent evidence for the presence of greater IRF-related transcriptional activity among up-regulated genes. IRF transcriptional activity has been previously linked to up-regulated genes among women with breast cancer who have a history of childhood maltreatment while accounting for depressive symptoms [19]. The present study extends this finding to adolescents and confirms that this differentiated immunological activity occurs independent of MDD. Among its many immune-activating functions, IRF enables macrophages to secrete superoxide anions that help to kill foreign cells [27]. Perhaps related to this essential host defense process, this data also implicated NRF1 and MAF-related transcriptional activity in the up-regulated genes among maltreated youth. Importantly, MAF-family transcriptional activity has been implicated in the phenotypic shift in macrophage function during an acute inflammatory response [26,58]. More specifically, in response to acute bacterial infection, MAF activity facilitates the production of cytokines that recruit monocytes to the site of the infection and suppresses protection against oxidative stress. As this MAF activity degrades, macrophages shift their function back to protective antioxidant defense [58]. Both MAF-related and NRF1 transcription factors are involved in the regulation of oxidative stress [59,60], and could indicate compensatory transcriptional activity that warrants further investigation among adversity-exposed populations.

Contrary to previous studies in which maltreatment was associated with a greater prevalence of NF-κB among up-regulated genes [19,23], maltreatment in the present sample was associated with greater prevalence of NF-κB transcriptional activity among down-regulated genes. Aberrations in NF-κB transcriptional activity in immune cells have been implicated in the development and progression of cancer, as well as autoimmune and other inflammatory disorders. See Ref. [61] for a review on NF-κB transcription and its clinical implications. For this reason, NF-κB activity has been a common target in pharmacological interventions aiming to mitigate dysregulated inflammatory signaling [62]. The inverse association between maltreatment and NF-κB in the present sample relative to others may be a by-product of our younger sample and a sign that links between maltreatment and increased NF-κB activity are moderated by age. For example, in another previous study of adolescents, NF-κB activity was implicated in up-regulated genes within CD8 T cells but not CD4 helper T cells [23], whereas NF-κB activity was implicated more broadly in up-regulated genes in a sample of adults [24]. More studies are needed which characterize these processes within a lifespan developmental framework.

Maltreatment was not associated with greater abundance of CREB transcriptional activity among up-regulated or down-regulated genes after accounting for MDD. This was somewhat unexpected given that CREB has been implicated in the exaggerated inflammatory response to psychosocial stress observed among adolescents exposed to adversity [25]. CREB is involved in the *β*-adrenergic modulation of immune activity by the sympathetic nervous system and includes cytokine production as well as cell differentiation, proliferation, and survival [63]. Together, the present data may indicate that CREB plays a role in robust acute immune reactivity that may be specific to psychosocial stress.

The results of this study must be considered in the context of the study’s limitations. First, while the study design aimed to disentangle childhood maltreatment from MDD, the four groups remained uneven such that adolescents exposed to severe childhood maltreatment who had no history of MDD or other psychiatric disorders were difficult to recruit despite the relatively young population and, therefore, under-represented relative to the other groups. Second, these data are also correlational and no causal inferences can be drawn. We cannot conclude from this data that adolescents exposed to maltreatment have larger non-classical CD16+ monocyte populations or that the transcriptional activity led to their differentiation into this cell subtype, only that the transcriptional activity among up- and down-regulated genes likely originated in this cell population. The disproportionate study of non-classical CD16+ monocytes in vitro has impeded basic understanding of their function in this regard [29]. More studies that track reactivity of these cells in vivo are needed e.g., [25]. Relatedly, inflammatory biology was measured using DBS. Results of genome-wide profiling of RNA from DBS relative to venous blood samples are largely concordant, *r* = 0.85 [47]. However, convergence or divergence of these observations with venous blood would be informative with respect to monocytes and macrophages in particular.

## 5. Conclusions

Overall, maltreatment was associated with differential immune cell activity whose differential gene expression largely originated in non-classical CD16+ monocytes, whose up-regulated genes were linked to IRF, NRF1, and MAF transcriptional activity and whose down-regulated genes were linked to NF-κβ. Taken together, maltreatment was related to a molecular signature that reflects innate immune activation that may favor host defense rather than quiescence and homeostasis, which may indirectly underlie the proinflammatory phenotype that has repeatedly been associated with this population and their lifelong experiences of health disparity.

## Figures and Tables

**Table 1:** Participant characteristics (*n* = 133).

		Maltreatment-Exposed (*n* = 46)		Not Maltreatment-Exposed (*n* = 87)		*p*-Value of Group Difference
Characteristic		*M* (*SD*)	% (*n*)	*M* (*SD*)	% (*n*)	
Age		15.52 (1.47)		15.36 (1.48)		*p* = 0.57
Tanner stage		4.34 (0.70)		4.22 (0.75)		*p* = 0.38
Female			73.9 (34)		77.0 (67)	*p* = 0.69
Race/Ethnicity ^1^						
	Hispanic/Latino		56.5 (26)		47.1 (41)	*p* = 0.30
	Non-Hispanic		43.5 (20)		52.9 (46)	
	American Indian or Alaska Native		6.5 (3)		3.4 (3)	*p* = 0.04
	Asian		0.0 (0)		13.8 (12)	
	Black or African American		19.6 (9)		9.1 (8)	
	Caucasian or White		41.3 (19)		50.6 (44)	
	Multi-racial		32.6 (15)		21.3 (19)	
	Native Hawaiian/Pacific Islander		0.0 (0)		1.1 (1)	
Body mass index (kg/m^2^)		25.77 (6.28)		22.92 (4.59)		*p* = 0.003
	Obese (BMI > 30)		21.7 (10)		9.2 (8)	*p* = 0.04
Ever smoked			2.2 (1)		6.9 (6)	*p* = 0.25
Current MDD			71.1 (33)		49.4 (43)	*p* = 0.01

Notes: ^1^ Groups not mutually exclusive.

**Table 2:** Differential transcriptional activity among maltreated adolescents with and without accounting for MDD.

	Before Accounting for MDD		After Accounting for MDD	
	*Mean (SE) log_2_ Ratio*	*p*	*Mean (SE) log_2_ Ratio*	*p*
IRF	0.03 (0.41)	0.94	0.78 (0.37)	0.036
NF-kB	−0.88 (0.25)	0.0007	−0.59 (0.23)	0.013
CREB	0.69 (0.26)	0.0087	0.41 (0.29)	0.16
MAF	0.91 (0.28)	0.0016	0.99 (0.39)	0.01
NRF1	0.34 (0.21)	0.11	0.63 (0.20)	0.0019

## Data Availability

Data from this study can be made available upon reasonable request.
